# Development and Validation of Ten-RNA Binding Protein Signature Predicts Overall Survival in Osteosarcoma

**DOI:** 10.3389/fmolb.2021.751842

**Published:** 2021-12-01

**Authors:** Jian Zhang, Xinxin Miao, Tianlong Wu, Jingyu Jia, Xigao Cheng

**Affiliations:** ^1^ Department of Orthopedics, The Second Affiliated Hospital of Nanchang University, Nanchang, China; ^2^ Institute of Orthopedics of Jiangxi Province, Nanchang, China; ^3^ Institute of Minimally Invasive Orthopedics, Nanchang University, Nanchang, China

**Keywords:** RNA-binding protein, osteosarcoma, prognostic signature, overall survival, nomogram

## Abstract

Osteosarcoma is a malignant tumor that originates in the bones with the characteristics of high malignancy, predisposition to metastasis, and poor prognosis. RNA binding proteins (RBPs) are closely related to various tumors, but their relationship with osteosarcoma remains unclear. Based on GTEx and TARGET RNA sequencing data, we applied differential analysis to obtain RBP genes that are differentially expressed in osteosarcoma, and analyzed the functions of these RBPs. After applying univariate and LASSO Cox regression analysis, 10 key prognostic RBPs (TDRD6, TLR8, NXT2, EIF4E3, RPS27L, CPEB3, RBM34, TERT, RPS29, and ZC3HAV1) were screened, and an RBP prognostic risk assessment model for patients with osteosarcoma was established. The independent cohort GSE21257 was used for external verification, and the results showed that the signature has an excellent ability to predict prognosis. In addition, a nomogram that can be used for clinical evaluation was constructed. Finally, the expression levels of 10 prognostic RBPs in osteosarcoma cells and tissues were confirmed through experiments. Our study identified a ten-gene prognostic marker related to RBP, which is of great significance for adjusting the treatment strategy of patients with osteosarcoma and exploring prognostic markers.

## Introduction

Osteosarcoma is most common in adolescents (Arndt and Crist, 1999; Gianferante et al., 2017). It is highly malignant, progresses quickly, and has a poor prognosis, which seriously affects family and social health (Stiller et al., 2006). Early treatment of osteosarcoma was mostly based on amputation, but the prognosis was poor (Fletcher et al., 2002). Subsequent chemotherapy improved the patient’s prognosis (Ritter and Bielack, 2010). So far, surgery combined with chemotherapy has become an effective method for the treatment of osteosarcoma. However, the 5 years survival rate of patients with metastatic osteosarcoma is <20% (Kempf-Bielack et al., 2005). Therefore, research to find new treatments to improve the prognosis of osteosarcoma patients is ongoing (Kansara et al., 2014).

RNA binding proteins (RBPs) are important molecules with RNA binding domains that are widely expressed in organisms (Lunde et al., 2007). RBPs combine with their targeted mRNA to form a ribonucleoprotein (RNP) complex, and regulate genes at the post-transcriptional level by various mechanisms, thereby rapidly and effectively changing mRNA expression (Gerstberger et al., 2014). In the past few decades, many studies have revealed that RBPs are abnormally expressed in tumors, affecting the conversion of mRNA to protein and participating in tumor occurrence (Chatterji and Rustgi, 2018; Moore et al., 2018; Wang et al., 2018). The RBP CPEB4 has an important connection with the progress of liver cancer, melanoma, and pancreatic cancer (Ortiz-Zapater et al., 2011; Calderone et al., 2016; Pérez-Guijarro et al., 2016). In addition, the RBP Musashi has a cancer-promoting effect in several cancer types, including medulloblastoma (Vo et al., 2012; Kudinov et al., 2017) and colorectal cancer (Li et al., 2015; Wang et al., 2015). Although more and more studies have proved that RBPs are directly involved in the occurrence and development of many tumors, their relationship with osteosarcoma remains unclear, and there is no reliable RBP-related prognostic signature for osteosarcoma.

In this study, we extracted transcriptome sequencing information from TARGET and GTEx databases, and screened RBP genes related to the prognosis of osteosarcoma, an RBP-related risk model was constructed in the TARGET database, and its predictive ability in osteosarcoma was verified. Our research may provide valuable molecular targets for the future treatment and prognosis of osteosarcoma and open up new ideas for research into RBPs in osteosarcoma.

## Materials and Methods

### Data Collection and Processing

The gene sequencing data and clinical information of 84 osteosarcoma patients were screened from the TARGET database (https://ocg.cancer.gov/programs/target). Gene expression data of musculoskeletal samples from 396 healthy humans were collected from the GTEx database (https://gtexportal.org/). To eliminate the platform data difference between TCGA and GTEx databases, the gene transcriptional expression data of each sample were transformed into log2 (FPKM value +1). Subsequently, the combat function from the “sva” R package was used to integrate the GTEx and TARGET datasets into one dataset.

The microarray dataset, named the GSE21257, was obtained from the high-throughput microarray expression profile database (Gene Expression Omnibus database, GEO, https://www.ncbi.nlm.nih.gov/geo/). GSE21257 contained the gene expression data and related clinical information of 53 osteosarcoma patients, which were used as the verification cohort for follow-up analysis and model verification.

**FIGURE 1:**
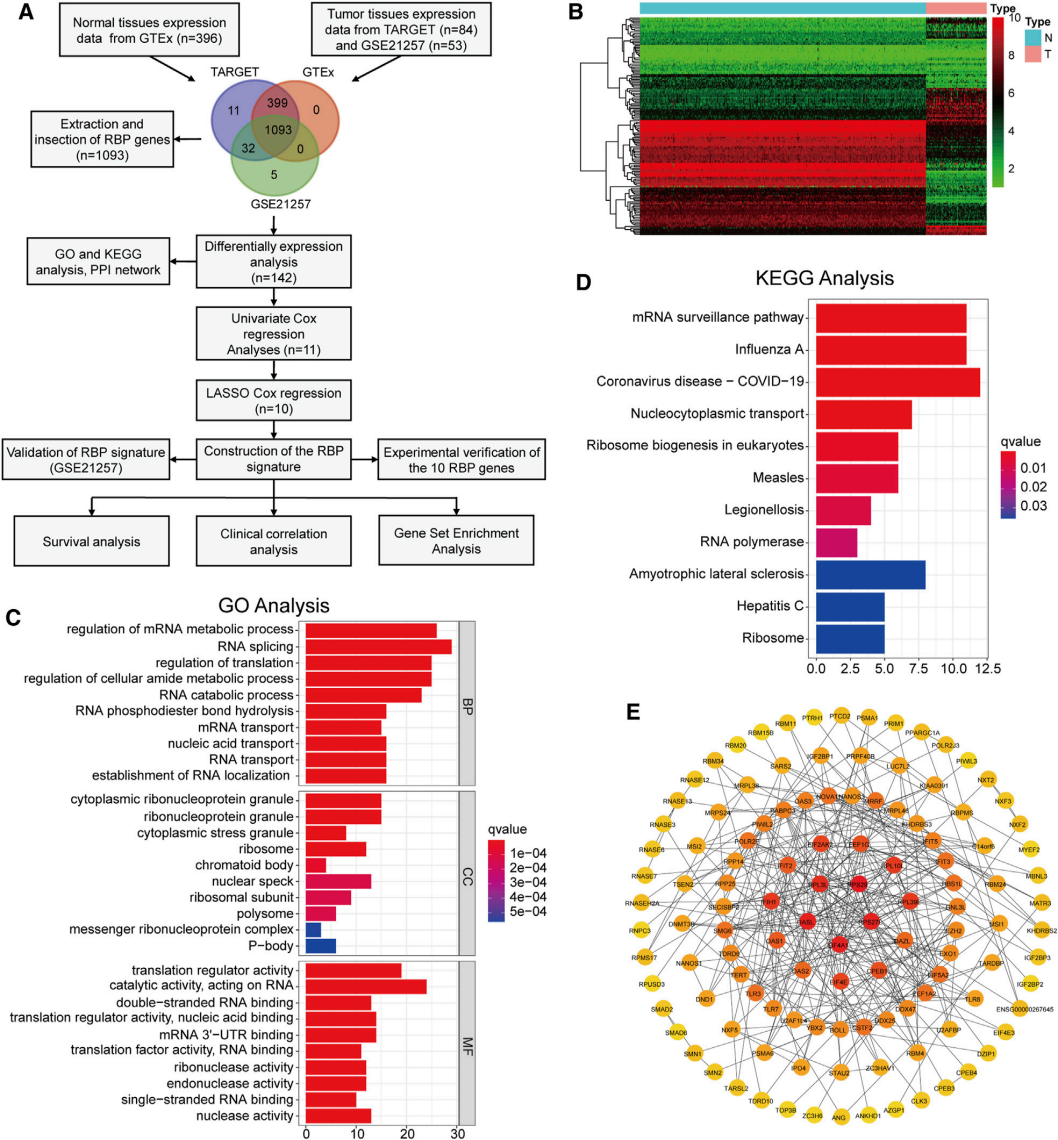
Identification of differentially expressed RBPs and functional analysis **(A)** The flow chart of this study **(B)** The heatmap of 142 differentially expressed RBPs **(C,D)** GO analysis and KEGG pathway analysis of the differentially expressed RBPs **(E)** PPI network diagram composed of differentially expressed RBPs.

The RNA-seq data was converted from FPKM to transcripts per million (TPM) using the algorithm described in the previous study for subsequent analysis (Li et al., 2010; Wagner et al., 2012). The 1,542 RBPs were obtained from previously published research (Gerstberger et al., 2014). The research process is shown in Figure 1A.

### Identification of Differentially Expressed RBPs (DERBPs) and Functional Enrichment Analysis

The “limma” package was used to screen the RBPs that were differentially expressed between osteosarcoma and normal tissues. The false discovery rate (FDR) < 0.05 and log2 fold change >1 were set as critical values. Then Gene Ontology (GO) function enrichment and Kyoto Encyclopedia of Genes and Genomes (KEGG) signaling pathway analyses were performed to explore the potential molecular mechanisms of the DERBPs. Analyzed the interaction between the DERBPs by the STRING online tool (http://www.string-db.org/) (Udhaya Kumar et al., 2020). Cytoscape software (version 3.7.2) was used to construct and visualize the PPI network. The expression levels of ZC3HAV1 in several common cancers were visualized by using the TIMER online tool (https://cistrome.shinyapps.io/timer/) and GEPIA online tool (Gene Expression Profiling Interactive Analysis, http://gepia.cancer-pku.cn/) (Kumar et al., 2019).

### Identification and Construction of Prognostic Signatures

Taking the TARGET dataset as the training cohort, the “survival” package in R was used to perform univariate Cox regression analysis to screen genes related to prognosis, which were then used as candidate genes for constructing the model. Based on the above-mentioned prognostic-related genes, we performed LASSO Cox regression analysis through the “glmnet” package to determine the best penalty value, and the corresponding gene was selected as the modeling gene. The formula for calculating the risk value of each patient was:

$$Risk\;score=\sum_{i=1}^{n}\left(Coef_{i}*x_{i}\right)$$

Here, $Coef_{i}\;$ represents the RBPs coefficient, and $x_{i}$ represents the RBPs expression.

### Evaluation and Verification of the Prognostic Signature

The above formula was used to calculate the risk score of each patient and divide them into high-risk groups and low-risk groups accordingly. Using the “survminer” package in R, we drawn the Kaplan–Meier (KM) curve. The “ggplot2” package was used to draw the distribution map of the risk score and survival status of the two patient groups and the modeled gene expression heatmap. The time-dependent ROC curve was produced through the “survivalROC” package in R. In parallel, combined with clinical information, such as age, gender, and tumor metastasis of osteosarcoma patients in the training cohort, the univariate and multivariate Cox regression model was used to analyze whether the risk score was an independent factor for judging the poor prognosis of osteosarcoma. The “rms” package in R was used to build and verify the nomogram model. The “calibrate” function of the rms software package was used to draw the calibration curve.

### Gene Set Enrichment Analysis (GSEA)

To verify the functional differences between the low-risk and high-risk groups, we used GSEA to compare the enrichment of tumor characteristic gene sets (hallmark gene sets) between the two groups. The tumor characteristic gene set comes from the Molecular Signatures database (MSigDB) database and contains 50 sets of genes involved in inflammation and hypoxia.

### Cell Lines and Cell Culture

The osteosarcoma cell lines (U2OS, 143B) and the osteoblast hFOB 1.19 cell line were purchased from the Cell Bank of the Chinese Academy of Sciences (Shanghai, China). The cells were grown in DMEM medium containing 10% fetal bovine serum (Gibco, United States) and 1% penicillin/streptomycin (solarbio, China). Osteosarcoma cells were grown at 37°C and 5% CO_2_. Osteoblasts were cultured at 34°C with a volume fraction of 5% CO_2_.

### Clinical Specimens

We collected six osteosarcoma tissues and six matched adjacent normal tissues. The samples came from patients who underwent surgery at The Second Affiliated Hospital of Nanchang University and were pathologically diagnosed with osteosarcoma. After the surgical resection, the sample is immediately stored in liquid nitrogen until the RNA or protein is extracted. All patients signed an informed consent form, and the study was approved by the Research Ethics Committee of the Second Affiliated Hospital of Nanchang University.

### RNA Extraction and Quantitative Real-Time PCR

Using TRIzol™ Reagent (Thermo Fisher Scientific, United States) to extract total RNA from cells and tissues, and using PrimeScript™ RT reagent Kit (Perfect Real Time) reverse transcription kit (Takara, Japan) to reverse transcription into cDNA, GAPDH as internal control, and real-time PCR was performed. According to the 2^−ΔΔCt^ method for relative quantitative analysis. The primer sequence was shown in Supplementary Table S1.

### Western Blotting

RIPA lysis buffer (Beyotime, China) was used to extract proteins from cells and osteosarcoma tissues. The equal amount of protein was separated by 10% SDS-PAGE gel electrophoresis, and then transferred to PVDF membrane by electroblotting (BioRad, United States). Then it was blocked with 5% skim milk, and incubated overnight with anti-ZC3HAV1 (1:1,000, Proteintech, China) and anti-GAPDH (1:2000, Proteintech, China) primary antibodies at 4 °C. After washing with TBST, incubated with secondary antibody (1:2000, Proteintech, china) for 1 h at room temperature. The blot was observed using ECL (enhanced chemiluminescence) and analyzed using ImageJ software.

### Statistical Analysis

All statistical analyses were conducted using R. 4.0.4 (https://www.r-project.org/) and SPSS Statistics 25 (https://www.ibm.com/products/software). Two groups were compared with Student’s t-tests. *p*-value < 0.05 is considered to be statistically significant.

## Results

### Screening and Functional Analysis of DERBPs

We extracted the RBPs expression matrix from the three datasets, and the Venn diagram displayed 1,093 intersected RBPs in all datasets (Figure 1A). Based on the dataset merged by TARGET and GTEx, we identified 142 DERBPs using the “limma” package and visualized by heatmap (Figure 1B). Next, functional analysis was performed on these DERBPs. GO analysis showed that, in terms of biological process (BP), DERBPs were mainly enriched in RNA splicing, regulation of translation, and mRNA metabolic process. In terms of cellular component (CC), cytoplasmic ribonucleoprotein granule, ribosome, and nuclear speck were enriched. In terms of molecular function (MF), DERBPs were mainly related to translation regulator activity, acting on RNA catalytic activity, and mRNA 3′-UTR binding (Figure 1C). Enrichment analysis of the KEGG pathway showed that the DERBPs mainly act on RNA transport (Figure 1D). We also constructed a PPI network. Figure 1E shows the interaction between the DERBPs. The darker the node, the more interacting proteins.

### Establishment of RBPs Prognostic Signature

**FIGURE 2:**
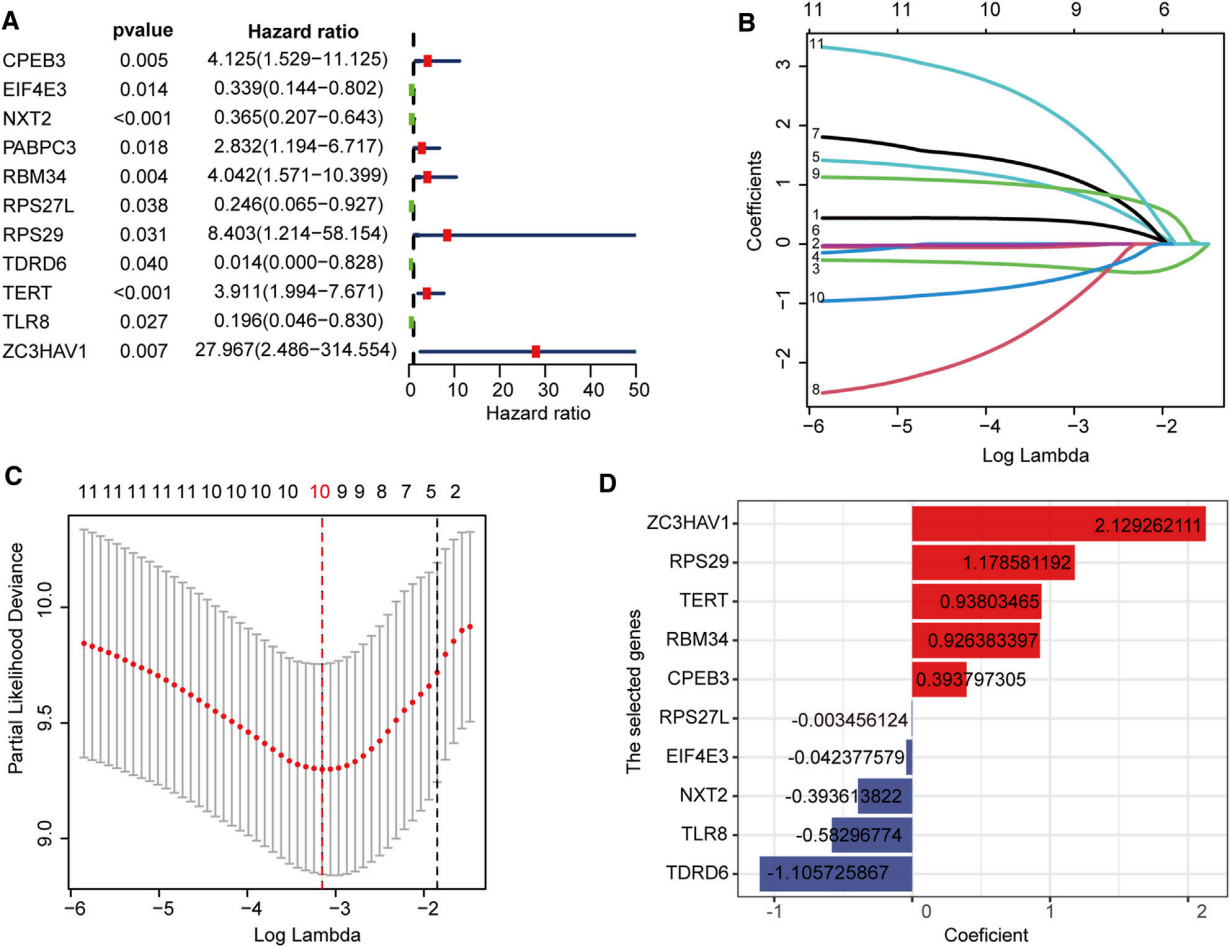
Construction of the RBPs prognostic signature **(A)** Univariate Cox regression revealed 11 RBPs associated with the prognosis of osteosarcoma **(B,C)** LASSO Cox regression analysis screened out 10 best genes to build the prognostic signature **(D)** The coefficients of 10 prognostic-related RBPs.

First, in the TARGET cohort, 142 DERBPs were analyzed using univariate regression analysis to obtain 11 RBPs related to prognosis (Figure 2A). LASSO regression analysis was performed on these 11 RBP genes (Figures 2B,C). When the log lambda reached the minimum, the parameters corresponding to the best modeling parameters and 10 model genes (TDRD6, TLR8, NXT2, EIF4E3, RPS27L, CPEB3, RBM34, TERT, RPS29, and ZC3HAV1), and their coefficients were obtained (Figure 2D).

### Evaluation and Verification of the RBPs Prognostic Signature

**FIGURE 3:**
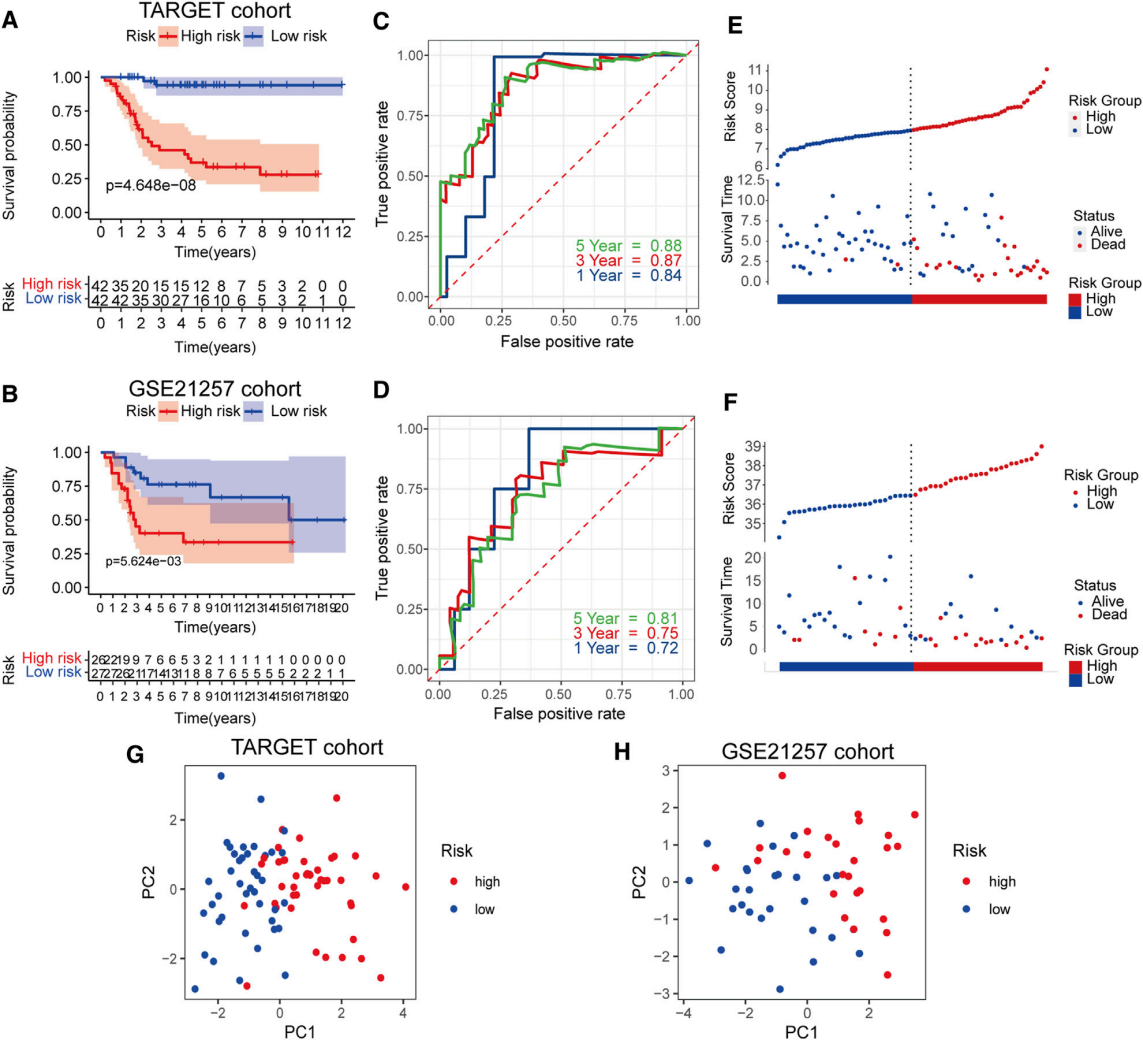
Survival analysis of RBPs prognostic signature in TARGET cohort and validation in GSE21257 cohort **(A,B)** Kaplan-Meier curves in the two cohorts **(C,D)** Risk score analysis of the signature in the two cohorts **(E,F)** The AUC for the prediction of 1, 3, 5 years survival rate **(G,H)** PCA based on the RBP-related signature.

We evaluated and verified the signature in the TARGET cohort and the GSE21257 cohort. Survival analysis showed a significant difference in survival between the high- and low-risk groups of the training and validation datasets (Figures 3A,B). For predictions of 1–5 years survival, the AUC values ​​in the training and test cohorts were 0.84, 0.87, and 0.88 and 0.72, 0.75, and 0.81, respectively (Figures 3C,D). As the risk score increased, the number of deaths gradually increased, and the survival time was significantly reduced (Figures 3E,F). PCA analysis showed that the distribution patterns of patients in different risk groups were significantly different (Figures 3G,H). These results show that the RBP signature has excellent forecasting capabilities for patient prognosis and has been verified in independent data.

### Relationship Between Risk Model and Clinical Characteristics

**FIGURE 4:**
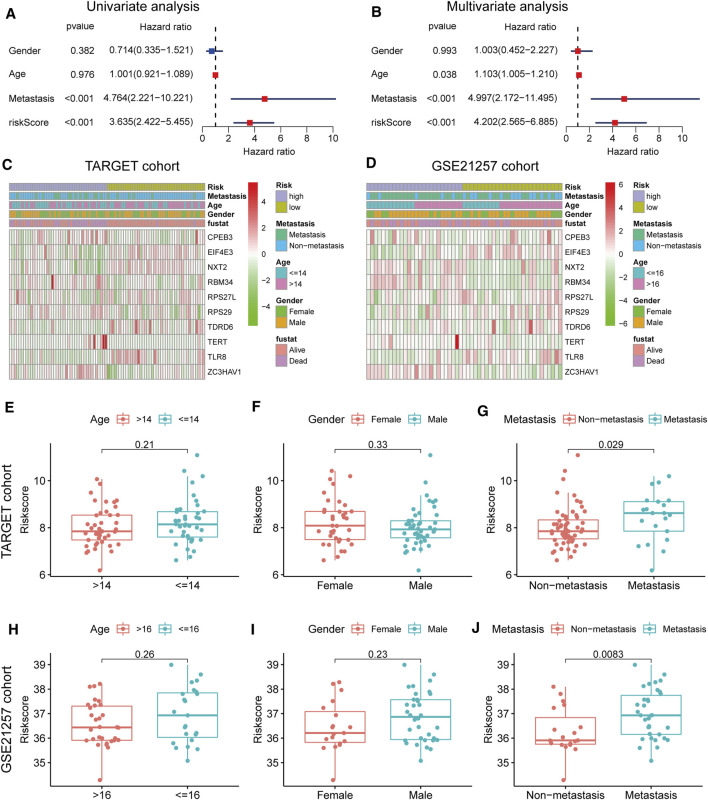
Clinical correlation analyses **(A,B)** Univariate and multivariate cox analysis showed the RBP genes signature and metastasis were two independent predictors of prognosis in osteosarcoma **(C,D)** The heatmap of the expression pattern of RBP is associated with the RBP signature and other clinical features **(E–G)** Relationship between risk score and clinical pathological factor in the TARGET cohort **(H–J)** Relationship between risk score and clinical pathological factor in the GSE21257 cohort.

In the TARGET cohort, univariate and multivariate Cox regression analysis showed that gender and age do not predict the prognosis of osteosarcoma patients, although metastasis status and risk score can be used as an independent factor in prognosis (Figures 4A,B). The difference in clinical characteristics and RBP gene expression patterns between the two groups is revealed by the heatmap (Figures 4C,D). The risk score was significantly related to metastasis, and high-risk patients were more likely to have tumor metastasis, as shown by the box plot in Figures 4E–J.

### Establishment of a Clinical Nomogram

**FIGURE 5:**
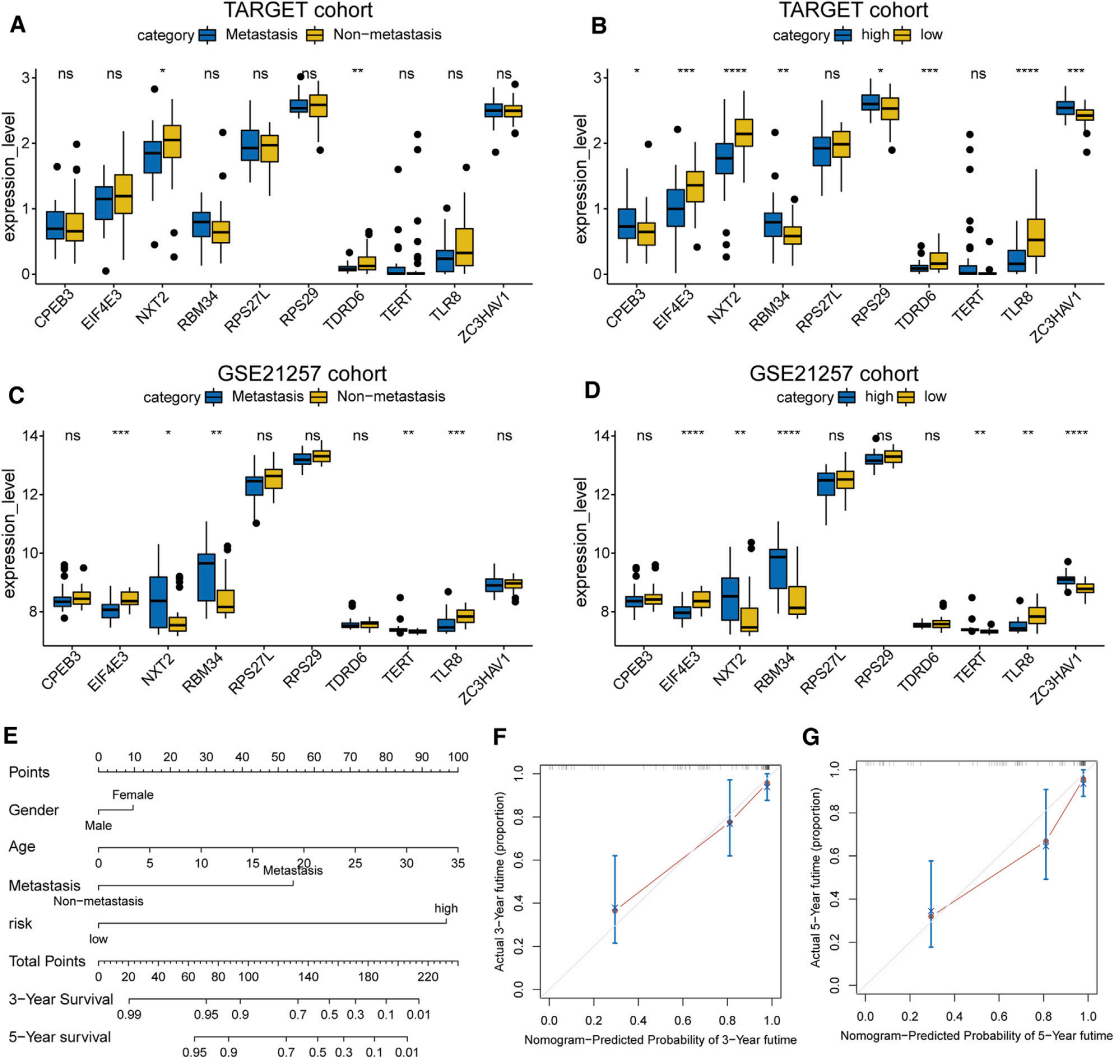
**(A–D)** The expression level of the 10 RBP genes, the patients were grouped according to metastasis and risk score **(E)** Nomogram based on gender, age, metastasis and risk in the TARGET cohort **(F,G)** Calibration plots of the nomogram for predicting the 3 and 5 years survival of osteosarcoma.

The expression levels of the 10 prognostic RBPs were displayed as boxplots, grouped according to the status of metastasis and risk (Figures 5A–D). Most RBPs showed obvious differential expression between the two groups, and the trends were consistent in the training and validation cohorts. We also established a clinical nomogram to clinically predict the survival of patients (Figure 5E). The C-index was used to evaluate the nomograms of the two datasets, and both sets of results showed robust predictive power (the C index of the TARGET dataset was 0.86, and the GSE21257 dataset was 0.74). A calibration plot showed that the predicted 3–5 years overall survival was in good agreement with the overall survival observed in the TARGET cohort (Figures 5F,G) and GSE21257 cohort (Supplementary Figure S1A,B). These data indicate that the nomogram is stable in predicting the survival of patients with osteosarcoma.

### Signal Pathway Enrichment Analysis of Characteristic Gene Sets

**FIGURE 6:**
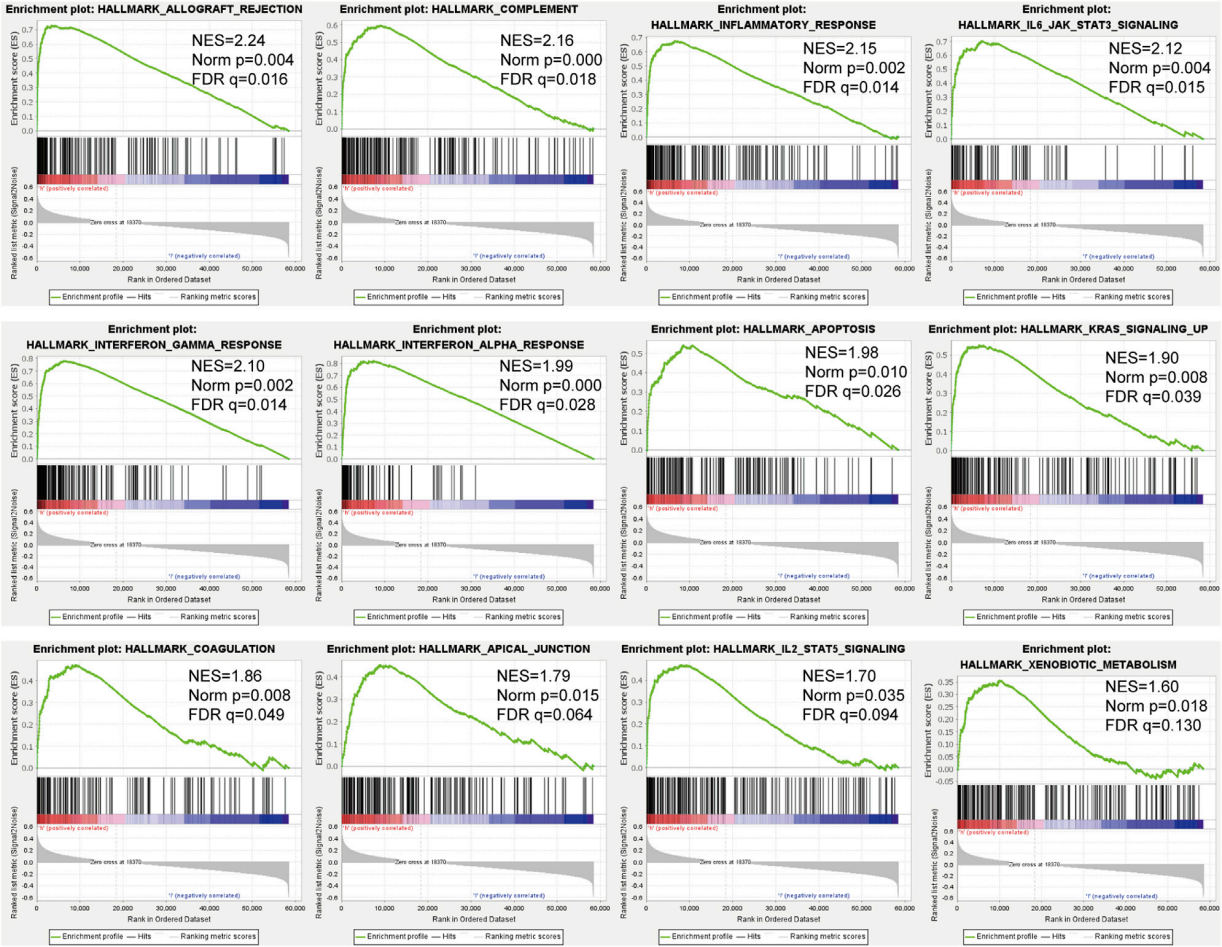
GSEA of osteosarcoma patients based on the RBPs prognostic signature in the TARGET cohort.

GSEA results showed that in the TARGET dataset, the signal pathways or biological processes related to the tumor and immunity were enriched in the high-risk group. Such as allograft rejection, complement, inflammatory response, IL6-JAK-STAT3 signaling, and the interferon-gamma response had higher negative enrichment scores (NES) in the high-risk groups (Figure 6).

### Verification of the Expression Level of RBP-Related Prognostic Genes

**FIGURE 7:**
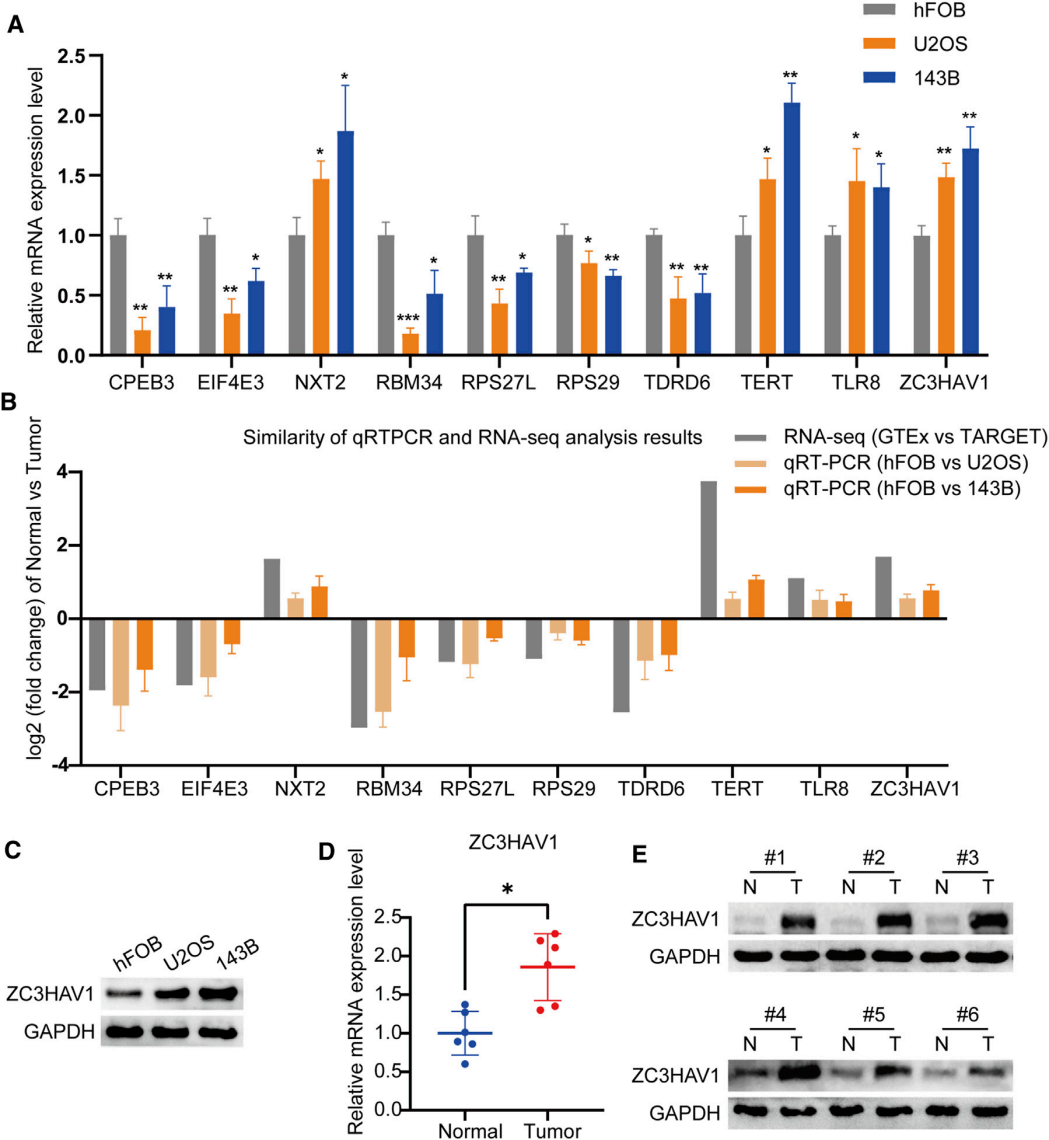
The expression levels of the prognostic RBP genes **(A)** The qRT-PCR result of the 10 RBP genes was evaluated by the 2-ΔΔCT method **(B)** The similarity of qRT-PCR and RNA-sequence analysis results of the 10 RBP genes. The data are expressed as mean ± standard deviation **(C)** The protein expression of ZC3HAV1 in osteoblast cell line and osteosarcoma cell lines **(D,E)** The mRNA and protein expression of ZC3HAV1 in six paired osteosarcoma tissues and adjacent normal tissues. *p < 0.05, **p < 0.01 and ***p < 0.001.

We verified the expression levels of these prognostic RBPs genes, by using qRT-PCR and western blot. Our results showed that compared with osteoblasts, CPEB3, EIF4E3, RBM34, RPS27L, RPS29 and TDRD6 were down-regulated in U2OS and 143B, while NXT2, TERT, TLR8 and ZC3HAV1 were up-regulated in osteosarcoma cells (Figure 7A). The RT-qPCR results of the 10 prognostic RBPs genes expression levels are consistent with the RNA-sequence data (Figure 7B). Among these 10 RBPs, ZC3HAV1 is an antiviral protein, recent studies have shown that it is related to the occurrence of a variety of cancers (Lin et al., 2014; Todorova et al., 2015). It is up-regulated in breast cancer, cervical squamous cell carcinoma, cholangiocarcinoma, esophageal cancer, glioma, head and neck squamous cell carcinoma and other malignant tumors (Supplementary Figure S1C, D). However, the role of ZC3HAV1 in osteosarcoma has not been reported yet. Therefore, we further quantified the expression of ZC3HAV1 in normal osteoblasts (hFOB1.19 cells) and 143B and U2OS osteosarcoma cell lines. Western blotting showed that compared with normal osteoblasts, ZC3HAV1 protein levels in osteosarcoma cells were significantly up-regulated (Figure 7C). Consistently, ZC3HAV1 mRNA and protein levels in osteosarcoma tissues were significantly higher than adjacent normal tissues (Figures 7D,E).

## Discussion

With the vigorous development of biotechnology and bioinformatics, genome analysis and various bioinformatics tools have been widely used to find cancer biomarkers (Tu et al., 2020; Udhaya Kumar et al., 2021). RBPs play a key role in regulating various RNA processes (Köster et al., 2017). Recent studies have shown that RBPs are not only involved in normal cell functions but are also major participants in the development and spread of tumors and so have great potential for the treatment of cancer (Pereira et al., 2017; Mohibi et al., 2019). Therefore, it is of great significance to study the clinical value and potential molecular mechanisms of RBP-related genes in osteosarcoma.

Although several signatures have recently been developed that can predict patient prognosis (Guan et al., 2020; Li et al., 2021), there are still some shortcomings in experimental verification. In addition, these studies lack corresponding clinical relevance studies and analyses based on clinical information (age, gender, metastasis), which affects the widespread application of signatures. Our study identified 142 DERBPs between tumor tissues and normal tissues based on GTEx and TARGET RNA sequencing data. Following systematic analysis of relevant biological pathways, a PPI network of these DERBPs was constructed. Single-factor and LASSO Cox regression analysis of abnormally expressed RBPs, resulted in 10 key prognostic RBPs (TDRD6, TLR8, NXT2, EIF4E3, RPS27L, CPEB3, RBM34, TERT, RPS29, and ZC3HAV1), and an RBP prognostic risk assessment model for patients with osteosarcoma was successfully constructed. The model was verified in the independent dataset GSE21257, and the results showed that the model has a good ability to predict prognosis. The constructed nomogram further visually and quantitatively describes the 3–5 years survival rate of patients with osteosarcoma. These results indicate that the RBPs’ prognostic signature established in this study is of great significance for the adjustment of treatment strategies and the exploration of prognostic markers for patients with osteosarcoma.

We have identified 10 key prognostic RBP genes, some of them are closely related to tumors. Telomerase reverse transcriptase (TERT) is a part of telomerase closely related to cancer (Yuan et al., 2019), and higher TERT expression in tumors can predict the poor prognosis of various cancers (Liu et al., 2016; Barthel et al., 2017; Ma et al., 2019). Cytoplasmic polyadenylation element-binding protein 3 (CPEB3) is a sequence-specific RBP whose overexpression inhibits the proliferation and migration of tumor cells, which has been proven in many studies (Tang et al., 2017; Luo and Wang, 2020; Zhang et al., 2020; Zhong et al., 2020). Ribosomal protein S27-like (RPS27L) is an evolutionarily preserved ribosomal protein. Xiong et al. showed that RPS27L can regulate genome stability and has potential tumor suppressor functions (Xiong et al., 2014). Eukaryotic translation initiation factor 4E family member 3 (EIF4E3) is a transformation initiating factor that can act as a tumor suppressor (Osborne et al., 2013; Volpon et al., 2013). Toll-like receptor (TLR) is a critical component of the innate immune response, and TLR8 is one of its subtypes (Khan et al., 2016). The *in vivo* study by Li et al. clearly shows that the metabolic reprogramming of regulatory T (Treg) cells mediated by TLR8 enhances anti-tumor immunity (Li et al., 2019). The 40S RP S29 coded by the RPS29 gene is a component of the small 40S ribosomal subunit, which is essential for rRNA processing and ribosomal biological production (O'Donohue et al., 2010). Compared with normal tissues, its expression is down-regulated in head and neck squamous cell carcinoma (Lallemant et al., 2009). But so far, TDRD6, NXT2, RBM34 have not been described in cancer. Zinc finger CCCH-type containing, antiviral 1 (ZC3HAV1), also known as zinc-finger antiviral protein (ZAP), has been shown to limit the replication of certain viruses, thereby preventing virus-related cancers such as liver cancer (Mao et al., 2013) and leukemia (Gao et al., 2002). For the first time, we found that ZC3HAV1 was up-regulated in osteosarcoma cell lines and also osteosarcoma tissues. Our results provide new ideas for exploring the role of RBPs in the development of osteosarcoma and may provide valuable perspectives for future cancer diagnosis and treatment.

The results of GSEA showed that patients in the high-risk group had higher NES in biological functions such as allogeneic rejection, complement system, inflammation, and IL6-JAK-STAT3 signaling. Aguirre et al. showed that transplant rejection and cancer immunomodulation have overlapping or even mutually exclusive mechanisms of action (Aguirre et al., 2019). The complement system is an important part of the inflammatory response, and inflammation involves all stages of tumorigenesis and cancer progression (Coussens and Werb, 2002). Moreover, supplemental activation regulates the adaptive immune response and may play a role in regulating the response of T cells to tumors (Carroll and Isenman, 2012). The IL-6/JAK/STAT3 pathway plays a key role in the growth and development of many human cancers (Johnson et al., 2018), and elevated IL-6 levels stimulate the overactivation of JAK/STAT3 signaling, which is usually related to poor patient outcomes (Ludwig et al., 1991; Kusaba et al., 2006; Chen et al., 2013).

It must be noted, however, that our research has some limitations. First, there are currently few public gene expression databases that contain prognostic information for patients with osteosarcoma, resulting in a small sample size for our study. In the future, larger sample size will be needed to build a more accurate prognostic model. Second, the clinical information of the dataset is not complete, and richer clinical data is needed to evaluate the relationship between genes and the clinic. Finally, we did not use *in vitro* or *in vivo* experiments to thoroughly verify our findings, which means that the exact mechanism of ZC3HAV1 involved in osteosarcoma is still unclear. This is an important subject that requires further research. It is necessary to conduct research on tissue and cell-type specificity loss and function gain to deepen our understanding.

In summary, this study systematically explored the expression and prognostic value of RBPs in osteosarcoma. By constructing a prognostic model of 10 RBP genes, our study can positively guide the future treatment and prognosis of osteosarcoma. The results of this study provide clear evidence for revealing the pathogenesis of osteosarcoma, developing new diagnostic ideas, finding new therapeutic targets and prognostic molecular markers.

## Data Availability

The original contributions presented in the study are included in the article/Supplementary Material further inquiries can be directed to the corresponding author.
