# Supplementary material for: Development and Validation of Ten-RNA Binding Protein Signature Predicts Overall Survival in Osteosarcoma
Source: Front Mol Biosci. 2021 Dec 1;8:751842. doi: 10.3389/fmolb.2021.751842 (PMC8671810; doi:10.3389/fmolb.2021.751842)
Supplement: Supplementary file 1 [file Table1.DOCX]

**Supplementary Table 1. Primers used in this study.**

| Gene | Direction | Sequences (5’ to 3’) |
| --- | --- | --- |
| CPEB3 | Forward | TGGAGAACTCCTTAATGGATATGA |
|  | Reverse | AAGTTTATTCCCATGCGTCCT |
| EIF4E3 | Forward | GACCAGCCTGCCTTTGAGAT |
|  | Reverse | TTCCAAACTGTGGACGTGCT |
| NXT2 | Forward | TTGCCTTCTAGTGAGTTCCAGG |
|  | Reverse | TGTAGTTTGGGACTGAGTTGCT |
| RBM34 | Forward | TACAGGCTTGGACAGGTCG |
|  | Reverse | CGTACACGGGTTGAATCTGGG |
| RPS27L | Forward | GTGACGACCTACGCACACGA |
|  | Reverse | GTGCTGCTTCCTCCTGAAGG |
| RPS29 | Forward | CGCTCTTGTCGTGTCTGTTCA |
|  | Reverse | CCTTCGCGTACTGACGGAAA |
| TDRD6 | Forward | CACCCTGTATGGGGAAGATG |
|  | Reverse | GCTCTGAAGAAAGCGGTCAG |
| TERT | Forward | CCAAGTTCCTGCACTGGCTGA |
|  | Reverse | TTCCCGATGCTGCCTGAC |
| TLR8 | Forward | AGTTTCTCTTCTCGGCCACC |
|  | Reverse | ACATGTTTTCCATGTTTCTGTTGT |
| ZC3HAV1 | Forward | ATCCACCTCTGTTCTGTAG |
|  | Reverse | TCTTCTCCATACTGAATCCAT |
| GAPDH | Forward | GAAGGTCGGAGTCAACGG ATTTG |
|  | Reverse | ATGGCATGGACTGTGGTCATGAG |
