# Supplementary figures and images for: Development and Validation of Ten-RNA Binding Protein Signature Predicts Overall Survival in Osteosarcoma
Source: Front Mol Biosci. 2021 Dec 1;8:751842. doi: 10.3389/fmolb.2021.751842 (PMC8671810; doi:10.3389/fmolb.2021.751842)

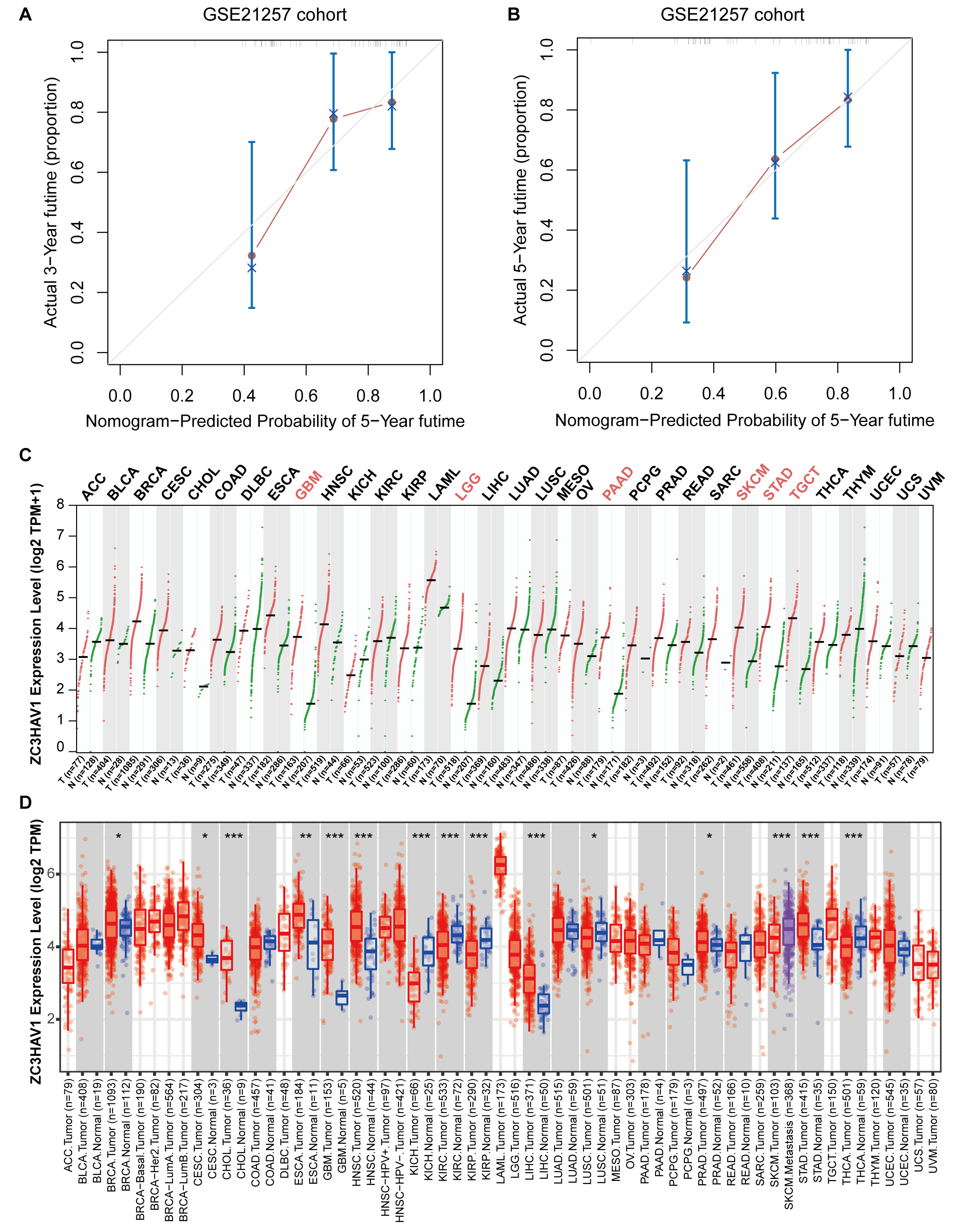

Supplement: Supplementary file 2 [file Image1.TIF]
